# Supplementary material for: What helps and hinders doctors in engaging in continuous professional development? An explanatory sequential design
Source: PLoS One. 2020 Aug 20;15(8):e0237632. doi: 10.1371/journal.pone.0237632 (PMC7446888; doi:10.1371/journal.pone.0237632)
Supplement: S1 File — (DOCX) [file pone.0237632.s001.docx]

**
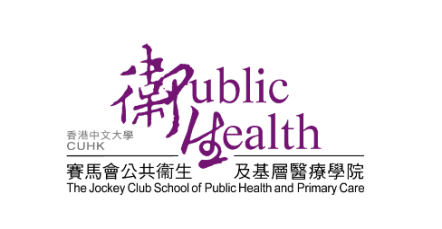

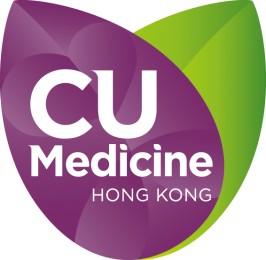

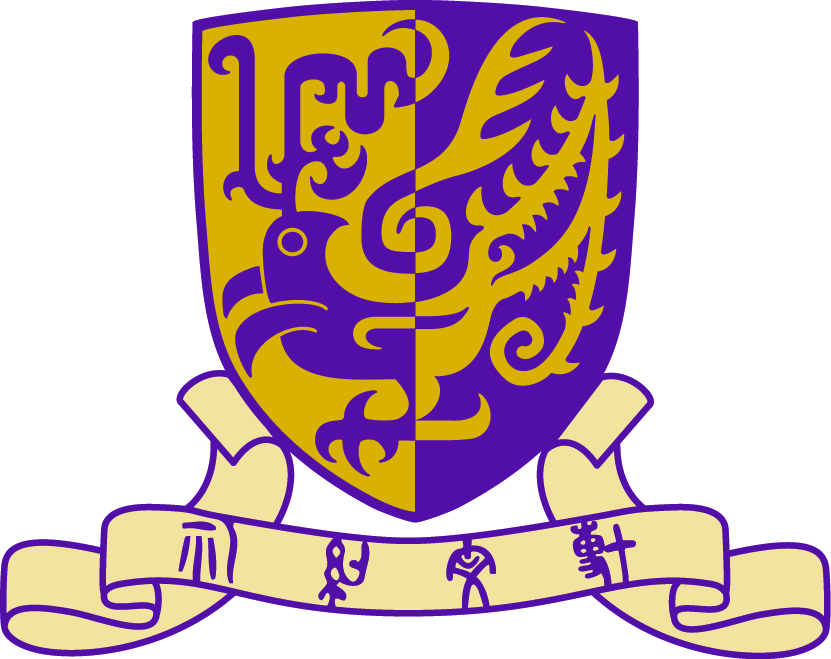
**

***The Chinese University of Hong Kong***

***Faculty of Medicine***

***The JC School of Public Health and Primary Care***

This survey is to explore doctors’ **perceived** **needs for continuous professional development**, and **attitudes towards medical regulation** in Hong Kong. Please answer all the questions and **return this Questionnaire together with a “Reply slip” either by post using the enclosed envelope or by fax at 2145 8517 on or before 15 June 2015.** All information provided will be kept strictly **confidential.**

| **Please write today’s date here:** | dd€€/mm€€/ 2015 |
| --- | --- |

*Please mark the box like this* ☑ *with a pen. Choose only ONE option unless specified otherwise.*

**PART 1: PERCEIVED NEEDS FOR CONTINUOUS PROFESSIONAL DEVELOPMENT**

| **Do you agree with the following statements?** | **Strongly Agree** | **Agree** | **Disagree** | **Strongly Disagree** |  | **Don’t know/ NA** |
| --- | --- | --- | --- | --- | --- | --- |
| (a) Advances in medical knowledge and technology require updating of knowledge and development of new skills for medical professionals. | □ | □ | □ | □ |  | □ |
| (b) I recognize my need to regularly update my medical knowledge. | □ | □ | □ | □ |  | □ |
| (c) I will fall behind in the standard of my professional practice if I stopped learning about new developments in my specialty/ area of practice. | □ | □ | □ | □ |  | □ |
| (d) All practicing doctors need to keep their professional knowledge updated. | □ | □ | □ | □ |  | □ |
| (e) **Have you participated in the following activity in the past one year?** | | | **Yes** | **No** |  | **NA** |
| (i) Self-learning e.g. reading journals/ articles | | | □ | □ |  |  |
| (ii) CME CPD activities not recognised by Medical Council of Hong Kong (MCHK) | | | □ | □ |  |  |
| (iii) CME CPD activities recognised by MCHK | | | □ | □ |  |  |
| (iv) ***For non-specialist:*** Enrolment in the MCHK CME Programme for Practising Doctors who are not taking CME Programme for Specialists | | | □ | □ |  | □ |
| (v) ***For specialist:*** Enrolment in the Hong Kong Academy of Medicine (HKAM) Colleges CMECPD Programmes | | | □ | □ |  | □ |
| (f) Do you encounter **any barriers to CME CPD learning**?  □A great deal □Significant □Few □No □Don’t know  *For those saying “a great deal”, “significant” and “few”,* ***what are the reasons****? (Multiple answers allowed)*  □Cost □Time □Not convenient □Work-life balance □No suitable activities  □Others, Pls specify ___________________________________ | | | | | | |
| **Do you agree with the following statements?** | **Strongly Agree** | **Agree** | **Disagree** | **Strongly Disagree** |  | **Don’t know/ NA** |
| (g) Generally, participation in CME CPD activities (which is recognized by MCHK/ HKAM) can improve my practical skills. | □ | □ | □ | □ |  | □ |
| (h) Generally, participation in CME CPD activities (which is recognized by MCHK/ HKAM) can improve patient outcome. | □ | □ | □ | □ |  | □ |
| (i) All doctors in Hong Kong should participate in CME CPD programme recognized by MCHK/ HKAM. | □ | □ | □ | □ |  | □ |
| (j) CME CPD should be required for all doctors in Hong Kong for renewal of practising certificates. | □ | □ | □ | □ |  | □ |
| (k) CME CPD should be included as one of the criteria for joining the government-initiated healthcare programmes e.g. vaccination programmes, elderly healthcare vouchers. | □ | □ | □ | □ |  | □ |

***Abbreviation:***

*CME – Continuing Medical Education; CPD – Continuous Professional Development; MCHK – Medical Council of Hong Kong Kong; HKAM – Hong Kong Academy of Medicine*

**PART 2: ATTITUDES TOWARDS MEDICAL REGULATION**

| (a) How **important are the following functions carried out by the Medical Council of Hong Kong (MCHK)** in order to assure public confidence in the medical profession? | | **Very important** | | **Important** | | **Not important** | | **Not at all important** | |  | | **Don’t know/ NA** |
| --- | --- | --- | --- | --- | --- | --- | --- | --- | --- | --- | --- | --- |
| (i) Quality assurance of pre-qualification education | □ | | □ | | □ | | □ | |  | | □ | |
| (ii) Ways of upholding professional standards and competence | □ | | □ | | □ | | □ | |  | | □ | |
| (iii) Mechanisms to detect professional misconduct and poor performance | □ | | □ | | □ | | □ | |  | | □ | |
| (iv) Fair disciplinary processes | □ | | □ | | □ | | □ | |  | | □ | |
| (v) Others: Please specify: | | | | | | | | | | | | |
| (b) In general, did you have **confidence in the MCHK** in | | **Very confident** | | **Confident** | | **Not confident** | | **Not at all confident** | |  | | **Don’t know/ NA** |
| (i) Maintaining doctors’ high professional standards | | □ | | □ | | □ | | □ | |  | | □ |
| (ii) Fostering doctors’ professional conduct | | □ | | □ | | □ | | □ | |  | | □ |
| (c) Currently MCHK has 28 members. 24 members are medical practitioners, and 4 members are lay members. Do you think **this structure is sufficient to assure public confidence in the medical regulation**?  □ Very sufficient □ Sufficient □ Insufficient □ Very insufficient □ Don’t know  *For those saying “insufficient” and “very insufficient”,* ***what are the reasons for insufficiency****? (Multiple answers allowed)*  □ Too few lay members □ Too many medical practitioners □ Not enough elected medical practitioners  □ No public/ patient representatives □ Others, Pls specify ___________________________________ | | | | | | | | | | | | |
| (d) How **important is each of the following processes in assuring a doctor’s competence?** | | **Very important** | | **Important** | | **Not important** | | **Not at all important** | |  | | **Don’t know/ NA** |
| (i) Taking part in the continuous medical education to keep up-to-date knowledge and skill | | □ | | □ | | □ | | □ | |  | | □ |
| (ii) Meeting certain performance assessment indicators e.g. high successful rate of treatments | | □ | | □ | | □ | | □ | |  | | □ |
| (iii) Receiving high ratings from healthcare professionals with whom they work | | □ | | □ | | □ | | □ | |  | | □ |
| (iv) Receiving high ratings from their patients | | □ | | □ | | □ | | □ | |  | | □ |
| (v) Being periodically assessed to show that they are currently competent to practice safely | | □ | | □ | | □ | | □ | |  | | □ |
| (vi) Others: Please specify: | | | | | | | | | | | | |

**PART 3: DEMORGRAPHICS**

(a) Your **gender**: □ Male □ Female

(b) Your **age**: □ 21-30 □ 31-40 □ 41-50 □ 51-60 □ 61-70 □ ≥71

(c) Are you a

- - **Non-specialist**
  - **Specialist, *please indicate the College(s) that you have registered:*** *(Multiple answers allowed)*

□[Anaesthesiologists](http://www.hkam.org.hk/hkamweb/pages_1_157.html) □[Community Medicine](http://www.hkam.org.hk/hkamweb/pages_1_158.html) □[Emergency Medicine](http://www.hkam.org.hk/hkamweb/pages_1_160.html) □[Family Physicians](http://www.hkam.org.hk/hkamweb/pages_1_161.html)

□[Obstetricians and Gynaecologists](http://www.hkam.org.hk/hkamweb/pages_1_162.html) □[Ophthalmologists](http://www.hkam.org.hk/hkamweb/pages_1_163.html) □[Orthopaedic Surgeons](http://www.hkam.org.hk/hkamweb/pages_1_164.html)

□[Otorhinolaryngologists](http://www.hkam.org.hk/hkamweb/pages_1_165.html) □[Paediatricians](http://www.hkam.org.hk/hkamweb/pages_1_166.html) □[Pathologists](http://www.hkam.org.hk/hkamweb/pages_1_167.html) □[Physicians](http://www.hkam.org.hk/hkamweb/pages_1_168.html) □[Psychiatrists](http://www.hkam.org.hk/hkamweb/pages_1_169.html)

□[Radiologists](http://www.hkam.org.hk/hkamweb/pages_1_170.html) □[Surgeons](http://www.hkam.org.hk/hkamweb/pages_1_171.html)  □ Others (Please Specify:_______________________)

(d) Your **main setting of current practice:** *(Multiple answers allowed)*

□ Hospital Authority □ Government □ Private Hospital □ Academic institution

□ Solo private practice □ Group private practice □ Others (Please Specify:_______________________)

***THANK YOU VERY MUCH!***

**Please return this questionnaire together with the “Reply slip” either by post using the enclosed envelope or by fax at 2145 8517 on or before 15 June 2015.**
