# Supplementary material for: What helps and hinders doctors in engaging in continuous professional development? An explanatory sequential design
Source: PLoS One. 2020 Aug 20;15(8):e0237632. doi: 10.1371/journal.pone.0237632 (PMC7446888; doi:10.1371/journal.pone.0237632)
Supplement: S2 File — (DOCX) [file pone.0237632.s002.docx]

**Semi-structured individual interviews**

- Do you think the current CME CPD programme in Hong Kong is adequate to ensure professional standards and competence? Why?
  - Types of activities
  - Format
  - Quality assurance
- How to enhance CME CPD programmes to ensure doctors’ competence?
- Do you encounter any barriers to CME CPD learning and how to overcome these barriers?
- Are there any methods for monitoring and assessing doctors’ performance in Hong Kong? Are they necessary?
- Would you recommend changes in the current system in monitoring and assessing doctors’ performance in Hong Kong?
