# Supplementary material for: What helps and hinders doctors in engaging in continuous professional development? An explanatory sequential design
Source: PLoS One. 2020 Aug 20;15(8):e0237632. doi: 10.1371/journal.pone.0237632 (PMC7446888; doi:10.1371/journal.pone.0237632)
Supplement: Attachment — Submitted filename: Response to Reviewers.docx [file pone.0237632.s004.docx]

**Response to Reviewers**

PONE-D-19-17445R1
What helps and hinders doctors in engaging in continuous professional development? An explanatory sequential design

**Comments**

Thank you for your responses and manuscript revisions. These have addressed most of the comments with some additional comments arising from the revised text (below). In addition while overall the language is acceptable however it would benefit from careful proofing to address grammatical and language issues.
***Responses:***

Thank you for your comments. We have proof-read the manuscript to correct the grammatical and language issues.

1. **The Abstract should be shortened to a maximum of 300 words**

***Responses:***

We have shortened the abstract to 300 words.

2. **Line 27 Introduces public expectations as a driver of the work however it is not clear what this expectation is and how it fits with CME and CPD; public expectation also do not seem to be a focus on the Introduction to the work.**

***Responses:***

Thank you for your comments. In Hong Kong, there is no mandated, structured and ongoing mechanism to assess all doctors’ performance and ensure their competence in Hong Kong. However, the public expect doctors to stay up-to-date and improve the safety and quality of healthcare services. Taking part in continuous professional development is one of the ways to maintain and improve practice of doctors. We agree public expectation is not the main focus for this work. Engaging doctors in continuous professional development is important for their professional development as well as patient safety due to advances in the medical technology and changes in clinical practice. We have revised this paragraph to make it clearer.

3. **The Introduction refers to both CME and CPD but does not differentiate between the two or the rationale for use of the terms singularly or in combination in this work. In many jurisdictions CPD is used as the umbrella term encompassing all post licensure learning needed to maintain clinical skills and knowledge. As the term used in MCHK is CME it is also unclear what respondents understood CME CPD to be as distinct from CME or CPD, or whether they understood the three terms to be synonymous with each other.**

***Responses:***

Thank you for your comments and apologize for not changing the terms used in the Hong Kong context. Many jurisdictions e.g. US and UK usually use continuous professional development (CPD) as the collective term to describe all post-licensure learning to maintain professional competence. However, in our local context, the Hong Kong Academy of Medicine has statutory responsibility for accreditation and training of all specialists. Continuous professional development for specialists is differentiated into components of Continuing Medical Education (CME) and Continuous Professional Development (CPD) with guidelines on the types and hours of programmes required for each of these 2 components required for renewal of specialist status. “CME CPD programmes” is the name which is used by the Hong Kong Academy of Medicine. The Hong Kong Medical Council is the statutory authority that licenses all doctors and registers specialists. Neither CME nor CPD is mandated for renewal of licenses. However, to encourage continuous medical education for non-specialists, the Medical Council of Hong Kong has launched a voluntary CME programme, and issues a “CME Certificate” to those doctors who fulfill the requirements. We have revised the text in using “CME CPD programmes” specifically for Hong Kong’s context, while we use the collective term “continuous professional development” for general purpose.

4. **A new section of text has been added between lines 208-218 discussing salaries however it is unclear how this relates to CME/CPD.**

***Responses:***

Thank you for your comments. This section is added in response to the reviewer’s comments in our first round of revision. The reviewer suggested to provide more background information of Hong Kong’s healthcare system (e.g. who are the specialists and their status and pay/ salary systems). This background information could facilitate the reader understand Hong Kong’s system, and appreciate the relatively high social status of doctors in Hong Kong. We have kept this information but have abbreviated the description.
